# Piscine orthoreovirus sequences in escaped farmed Atlantic salmon in Washington and British Columbia

**DOI:** 10.1186/s12985-019-1148-2

**Published:** 2019-04-02

**Authors:** Molly J. T. Kibenge, Yingwei Wang, Nick Gayeski, Alexandra Morton, Kurt Beardslee, Bill McMillan, Frederick S. B. Kibenge

**Affiliations:** 1 Department of Pathology and Microbiology, Atlantic Veterinary College, University of Prince Edward Island, 550 University Ave, Charlottetown, P.E.I C1A 4P3 Canada; 2 School of Mathematical and Computational Sciences, University of Prince Edward Island, 550 University Ave, Charlottetown, P.E.I C1A 4P3 Canada; 3 Wild Fish Conservancy, PO Box 402, 15629 Main St. NE, Duvall, WA 98019 USA; 4 Raincoast Research Society, Box 399, Sointula, BC V0N 3E0 Canada

**Keywords:** Piscine orthoreovirus, PRV, Emergent virus, Salmon aquaculture, *Reoviridae*, Heart and skeletal inflammation, HSMI, Farmed Atlantic salmon

## Abstract

**Background:**

Piscine orthoreovirus (PRV) is an emergent virus in salmon aquaculture belonging to the family *Reoviridae*. PRV is associated with a growing list of pathological conditions including heart and skeletal inflammation (HSMI) of farmed Atlantic salmon. Despite widespread PRV infection in commercially farmed Atlantic salmon, information on PRV prevalence and on the genetic sequence variation of PRV in Atlantic salmon on the north Pacific Coast is limited.

**Methods:**

Feral Atlantic salmon caught in Washington State and British Columbia following a large containment failure at a farm in northern Puget Sound were sampled. Fish tissues were tested for PRV by RT-qPCR assay for segment L1 and conventional RT-PCR for PRV segment S1. The PCR products were sequenced and their relationship to PRV strains in GenBank was determined using phylogenetic analysis and nucleotide and amino acid homology comparisons.

**Results:**

Following the escape of 253,000 Atlantic salmon from a salmon farm in Washington State, USA, 72/73 tissue samples from 27 Atlantic salmon captured shortly after the escape tested PRV-positive. We estimate PRV-prevalence in the source farm population at 95% or greater. The PRV found in the fish was identified as PRV sub-genotype Ia and very similar to PRV from farmed Atlantic salmon in Iceland. This correlates with the source of the fish in the farm. Eggs of infected fish were positive for PRV indicating the possibility of vertical transfer and spread with fish egg transports.

**Conclusions:**

PRV prevalence was close to 100% in farmed Atlantic salmon that were caught in Washington State and British Columbia following a large containment failure at a farm in northern Puget Sound. The PRV strains present in the escaped Atlantic salmon were very similar to the PRV strain reported in farmed Atlantic salmon from the source hatchery in Iceland that was used to stock commercial aquaculture sites in Washington State. This study emphasizes the need to screen Atlantic salmon broodstock for PRV, particularly where used to supply eggs to the global Atlantic salmon farming industry thereby improving our understanding of PRV epidemiology.

**Electronic supplementary material:**

The online version of this article (10.1186/s12985-019-1148-2) contains supplementary material, which is available to authorized users.

## Background

Piscine orthoreovirus (PRV) is a member of the *Reoviridae* family [1]. Reovirus particles have a segmented double-stranded RNA genome in a non-enveloped double capsid [2], which makes PRV relatively resistant to disinfection [3], and durable outside a host, allowing it to remain infectious, as it spreads via marine currents [4].

PRV is an emerging virus with new isolates being reported throughout the global salmon farming industry [5]. The PRV segment S1 sequence was initially used by Kibenge et al. [6] to group PRV isolates from Norway, Chile and BC-Canada into one genotype, genotype I, with two major sub-genotypes designated Ia and Ib. Both Ia and Ib sub-genotypes occur in Norway [4] and Chile, whereas only sub-genotype Ia was found in BC-Canada in farmed Atlantic salmon and wild Pacific salmon [6]. Godoy et al. [7] reported identification of a second genotype, genotype II, among PRV isolates from Norway and Chile. Soon after, another PRV (designated PRV-2) found in Japan was shown also to belong to genotype II [8].

PRV genotype I is associated with heart and skeletal muscle inflammation (HSMI) in farmed Atlantic salmon *Salmo salar* in Norway [9], Chile [7], and BC-Canada [10]. Additionally, while not yet identified as the causative agent, PRV is associated with the following syndromes: melanised foci in white muscle in Atlantic salmon in Norway [11], farmed Chinook salmon *Onchorhynchus tshawytscha* exhibiting jaundice syndrome in BC-Canada [12, 13], PRV genotype II (PRV-3) in Coho salmon *O. kisutchi* with HSMI-like disease in Chile [7] and rainbow trout *O. mykiss* in Norway [14, 15] Denmark, Scotland and Germany [16], and in wild and farmed brown trout *S. trutta* in Italy [16] and France (Bigarre 2016, unpublished observations). PRV genotype II (PRV-3) sequences have also been detected in rainbow trout affected by idiopathic syndrome of rainbow trout (ISRT) [17] and in a moribund Coho salmon with jaundice [18] in Chile. Another variant of PRV genotype II (PRV-2) is the etiologic agent of erythrocytic inclusion body syndrome (EIBS), a condition associated with mass mortality in juvenile Coho salmon in Japan [8].

PRV targets red blood cells in Atlantic salmon [19, 20]. The response of these cells to PRV-Ia has been found to cause distinctly different diseases in salmon from the Atlantic versus the Pacific [13]. PRV infection in Pacific salmon is associated with reduced upriver migration success [21, 22]. The BC-Canada strains of PRV found in farmed and wild salmon [22] are closely related to Norwegian PRV strains. While long-term presence of PRV in the eastern Pacific has been suggested [23, 24], the evidence that it was introduced from Norway is substantial [6, 25]. However, the evidence for vertical transmission of PRV is ambiguous [3, 26].

The largest number of farmed Atlantic salmon tested for PRV in BC-Canada found PRV in approximately 80% of 146 pooled samples from 539 Atlantic salmon tested in 2010 (Marty and Bidulka, 2013, unpublished observations). Purcell et al. [27] tested returning adult Pacific salmonids in Washington and Alaska for PRV, but not Atlantic salmon or other commercially cultured fish. Recently, Morton et al. [22] reported on prevalence of PRV in wild salmon exposed and unexposed to salmon farms including 262 fresh BC farmed Atlantic salmon purchased from supermarkets and 601 wild Pacific salmonids (*Oncorhynchus spp*.) sampled from marine and freshwater throughout southern BC-Canada. PRV was detected in 95% of farmed Atlantic salmon, 69% of farmed steelhead, 5–50% of wild salmon and 3–75% of trout in lakes [22].

**Fig. 1:**
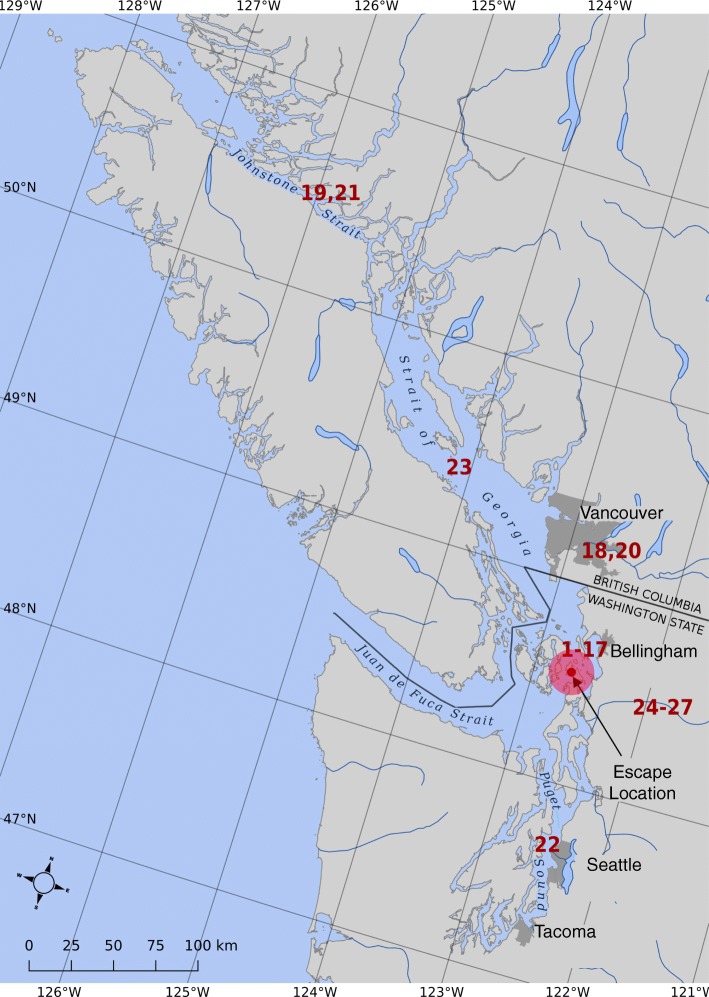
Map showing location of the farm where the escape occurred and capture site of the Atlantic salmon tested for PRV in this study using numbers assigned to each fish in Table 1

Currently, Atlantic salmon are commercially farmed in the coastal eastern Pacific Ocean with the majority of farms located in BC-Canada and a single company with eight sites in Puget Sound and the eastern Strait of Juan de Fuca in Washington State as of August 2017. On August 19, 2017, a catastrophic containment failure occurred at one of three farm sites in Deepwater Bay, collectively called the Cypress Island facility, in Northern Puget Sound (Fig. 1). The Washington State Departments of Ecology (DOE), Fish and Wildlife (DFW) and Natural Resources (DNR) estimated that 305,000 Atlantic salmon were in the farm net pen at the time of the failure of which fewer than 55,000 were recaptured in the immediate vicinity of the site by farm staff within 10–14 days of the escape. 253,000 +/− 10,000 were estimated to have escaped to marine waters of Puget Sound, the Strait of Juan de Fuca and Georgia Strait and to freshwater rivers discharging to these marine waters ([28], pp. 109–111, Table 4). Over 9,072 kg (20,000 pounds) of Atlantic salmon were caught in the weeks immediately following the escape [29]. Dozens of escaped Atlantic salmon were reported caught in the nearby Skagit River as far as 67 miles above saltwater through January, 2018 [29] (Bill McMillan personal observation). Beginning in September, 2017, Atlantic salmon were also caught in BC-Canada (Fig. 1). BC-Canada has approximately 120 licensed Atlantic salmon farm sites, and while large numbers of escaped Atlantic salmon have been caught in some years [30], none have been caught in recent years prior to the escape (data Alexandra Morton). In Norway, escaped farmed salmon, which are most often infected with PRV, could be a transmission vector for freshwater infections in wild fish if they enter rivers [31–33].

While the Norwegian-Mowi strain of Atlantic salmon is the predominate farm salmon in BC [34], the single company operating Atlantic salmon farms in Washington State imports Atlantic salmon eggs from Iceland [35]. PRV sub-genotype Ia is widespread in Iceland [36], although no sequence has been deposited in the GenBank database. The Washington State Departments of Ecology (DOE), Fish and Wildlife (DFW) and Natural Resources (DNR) report that 4 out of 4 of the escaped Atlantic salmon that they tested after the escape were positive for PRV ([28], 98, pp.).

The primary goals of the present study were to determine the prevalence of PRV in a subset of recaptured Atlantic salmon representative of the 305,000 that had been in the Cypress Island facility, and to identify the strains of PRV present in these fish and their genetic relationship to known PRV sequences.

## Methods

### Fish sampling and processing

The fish tissue source for all samples is detailed in Table 1.

**Table 1:** Summary of PRV testing by real-time, reverse transcriptase PCR (RT-qPCR) for all samples in this study

Collection Date	Location^1^	Fish ID	# on map^2^	Fish tissue	AVC #^3^	PRV Seg. L1 Cts	PRV Seg. S1 product^4^
Aug-27-2017	Home Port Bellingham-WA	WFC-2011-75	1	Gill	VT01192018–07	28.09	Positive
				Heart	VT01192018–08	31.74	
				Head kidney	VT01192018–09	28.85	
		WFC-2011-76	2	Gill	VT01192018–10	30.77	
				Heart	VT01192018–11	32.11	
				Head kidney	VT01192018–12	30.33	
		WFC-2011-77	3	Gill	VT01192018–13	29.65	
				Heart	VT01192018–14	30.52	
				Head kidney	VT01192018–15	28.63	
		WFC-2011-78	4	Gill	VT01192018–16	27.67	Positive
				Heart	VT01192018–17	30.96	
				Head kidney	VT01192018–18	28.67	
		WFC-2011-79	5	Gill	VT01192018–19	28.78	
				Heart	VT01192018–20	27.72	
				Head kidney	VT01192018–21	25.73	Positive
		WFC-2011-80	6	Gill	VT01192018–22	27.78	
				Heart	VT01192018–23	26.52	
				Head kidney	VT01192018–24	26.53	
		WFC-2011-81	7	Gill	VT01192018–25	29.33	
				Heart	VT01192018–26	26.94	Positive
				Head kidney	VT01192018–27	26.44	
		WFC-2011-82	8	Gill	VT01192018–28	26.15	
				Heart	VT01192018–29	26.98	
				Head kidney	VT01192018–30	29.91	
		WFC-2011-83	9	Gill	VT01192018–31	30.10	
				Heart	VT01192018–32	25.94	
				Head kidney	VT01192018–33	27.03	Positive
		WFC-2011-103	10	Gill	VT01192018–34	31.19	
				Heart	VT01192018–35	29.53	
				Head kidney	VT01192018–36	27.98	
		WFC-2011-104	11	Gill	VT01192018–37	31.68	
				Heart	VT01192018–38	28.91	
				Head kidney	VT01192018–39	30.12	
		WFC-2011-105	12	Gill	VT01192018–40	30.12	
				Heart	VT01192018–41	29.99	
				Head kidney	VT01192018–42	29.06	
		WFC-2011-106	13	Gill	VT01192018–43	24.89	Positive
				Heart	VT01192018–44	26.39	
				Head kidney	VT01192018–45	24.10	
Sept-11-2017	Home Port Bellingham-WA	WFC-2011-107	14	Gill	VT01192018–46	24.68	
				Heart	VT01192018–47	26.09	
				Head kidney	VT01192018–48	23.72	Positive
		WFC-2011-108	15	Gill	VT01192018–49	25.28	
				Heart	VT01192018–50	25.04	
				Head kidney	VT01192018–51	23.27	
		WFC-2011-109	16	Gill	VT01192018–52	23.60	Positive
				Heart	VT01192018–53	24.60	Positive
				Head kidney	VT01192018–54	27.32	
		WFC-2011-110	17	Gill	VT01192018–55	28.42	
				Heart	VT01192018–56	28.76	
				Head kidney	VT01192018–57	26.38	
Sept-14-2017	Lower Fraser River-BC	AE01	18	Gill, Heart	VT10042017–372	33.00	Negative
Oct-08-2017	Johnstone St-BC	AE02	19	Gill, Heart, Liver	VT10252017–405	27.33	Positive
Oct-10-2017	Lower Fraser River-BC	AE03	20	Gill, Heart, Liver	VT10252017–407	30.41	Positive
Oct-17-2017	Johnstone St-BC	AE04	21	Gill, Heart, Liver	VT10252017–408	32.04	
	St. of Juan De Fuca-WA	WFC-2011-13	22	Gill	VT01192018–01	0	
				Heart	VT01192018–02	35.9	
				Head kidney	VT01192018–03	34.10	
Nov-01-2017	Salish Sea-BC	AE05	23	Heart, Liver, Spleen, Head kidney	VT11022017–423	25.86	Positive
Dec-31-2017	Skagit River, Birdsview-WA	WFC-2011-14	24	Gill	VT01192018–04	35.60	
				Heart	VT01192018–05	33.47	
				Liver	VT01192018–06	34.74	
Jan-25-2018	Skagit River, mile 46-WA	WFC-2011-111	25	Gill	VT04202018–118	29.52	Negative
				Heart	VT04202018–119	25.97	Positive
				Liver	VT04202018–120	29.64	Positive
				Eggs	VT04202018–121	31.10	Positive
Jan-24-2018	Skagit River, mile 46-WA	WFC-2011-112	26	Gill	VT04202018–122	32.91	Negative
				Heart	VT04202018–123	29.76	Positive
				Liver	VT04202018–124	29.80	Positive
				Eggs	VT04202018–125	34.78	Negative
Jan-26-2018	Skagit River, mile 46-WA	WFC-2011-113	27	Gill	VT04202018–126	31.24	Negative
				Heart	VT04202018–127	28.76	Negative
				Liver	VT04202018–128	30.66	Negative
Apr-2018	Retail “Product of Iceland”	WFC-2011-114	28	Muscle	VT04202018–129	24.94	Positive

^1^Location source of samples included Home Port, Bellingham Bay, Washington (Home Port Bellingham-WA), Strait of Juan De Fuca, Port Townsend, Washington (Strait of Juan De Fuca-WA), Skagit River near Birdsview, Washington (Skagit River, Birdsview-WA), Skagit River at mile 46, Washington (Skagit River, mile 46-WA), Lower Fraser River, BC-Canada (Lower Fraser River-BC), Johnstone St., BC-Canada (Johnstone St-BC), Salish Sea, BC-Canada (Salish Sea-BC), and market-bought fish from a retail store in Redmond, Washington, labeled “Product of Iceland” (Retail “Product of Iceland”)

^2^# on map refers to the location source of the fish on the map shown as Fig. 1

^3^AVC # denotes accession number of sample at the testing laboratory at the Atlantic Veterinary College, University of Prince Edward Island

^4^Selected samples were tested in conventional RT-PCR for full-length PRV S1 segment. The presence of a 1100 base pair PCR product band in agarose gel is indicated as Positive . Negative denotes no band seen in agarose gel

*Washington State samples:* Tissue samples were obtained from 17 of the Atlantic salmon caught in the vicinity of the escape by gillnet and purse seine gear in the days following the August 19, 2017, escape (WFC-2011-75 – WFC-2011-110, Table 1). Tissues were also collected from: an Atlantic salmon captured in the eastern Strait of Juan de Fuca near Port Townsend, Washington (WFC-2011-13, Table 1), an angler-caught fish in the nearby Skagit River at river mile 46 (WFC-2011-14, Table 1) and three more fish captured in the Skagit River at river mile 46 in late January, 2018 (WFC-2011-111 – WFC-2011-114, Table 1). In addition, whole egg skeins were collected from two of the Atlantic salmon caught in late January, 2018 (WFC-2011-111, WFC-2011-112). One sample of farmed Atlantic salmon labeled “Product of Iceland” was obtained from a retail store in Redmond, Washington State.

All fish were euthanatized by percussion to the head and then placed on ice immediately after capture/purchase and were either frozen or placed on ice and sampled within 24 h. Samples of gill, heart, liver, head kidney, and/or muscle were individually preserved in RNALater™ (Ambion Inc). Prior to obtaining samples of eggs from each egg skein, each skein was individually rinsed in running tap water for 30 s to remove any surface fluids. Samples of eggs were then taken from the interior of each skein and placed in labeled vials containing RNALater™, as for organ tissue samples. The samples were then shipped under a Canadian Food Inspection Agency (CFIA) importation permit to the Atlantic Veterinary College at the University of Prince Edward Island for testing for PRV and other aquatic animal viruses.

*BC-Canada samples:* Five Atlantic salmon were captured from September 14 – November 1, 2017, in British Columbia between the lower Fraser River and Johnstone Strait (Table 1, Fig. 1). The commercial fishermen who caught them euthanatized the fish by percussion to the head and placed them on ice for 2–3 days. When received, samples of gill, heart, spleen, liver, and head kidney were pooled to loosely fill a 1.5 ml container of RNALater™ per fish, i.e., all tissue from each fish were combined into a single sample which was shipped on ice to the Atlantic Veterinary College at the University of Prince Edward Island.

At the testing lab, each sample was removed from the RNALater™ preservative and was weighed, chopped into small pieces and directly macerated to a 10% suspension (*w*/*v*) in L-15 medium (Winset Inc) with 10x antibiotics/antimycotic (Life Technologies Inc), except for egg samples which were directly macerated in Qiazol (QIAGEN), using Green MagnaLyser beads (Roche Diagnostics). The tissue homogenate was immediately used for total RNA extraction.

In the present study, there was no opportunity to collect back samples of Atlantic salmon organs or eggs from the same environment because the presence of escaped farmed Atlantic salmon in Washington State and BC-Canada was a transient event and did not appear in similar fisheries the following year, 2018.

### RNA extraction and RT-qPCR

Total RNA was isolated using a modified total RNA extraction protocol with Trizol Reagent (Life Technologies Inc) and RNeasy mini Kit (QIAGEN) as previously described [6]. Total RNA from eggs was extracted using the RNeasy Lipid Tissue mini kit (QIAGEN) which includes Qiazol. RT-qPCR was run on the LightCycler 480 (Roche Applied Science), version 4.0. The crossing point (Cp) or threshold cycle (Ct) was determined by use of the maximum-second-derivative function on the LightCycler software release 1.5.0. The OneStep RT-PCR kit (QIAGEN) was employed for all RT-qPCR reactions according to the manufacturer’s specifications. Sample RNA quality was based on RT-qPCR for elongation factor 1 alpha (ELF-1α) as internal control targeting Atlantic salmon ELF-1α carried out using Roche LightCycler 480 RNA master Hydrolysis Probe kit (Roche Diagnostics). The primers and probes and reaction conditions used were as previously described [37] and were designed to specifically amplify and detect selected ELF-1α coding sequences in Atlantic salmon, Coho salmon and rainbow trout [37]. The ELF-1α assay was performed in 2 wells per sample, and a sample with Ct value of ≤22.0 was considered of acceptable RNA quality [38]. The RT-qPCR assay for PRV used the primer-probe set sequences and reaction conditions as previously described [6] with minor modifications. Total RNA was denatured at 95 °C for 5 min and immediately placed on ice before use in the RT-qPCR. The PRV assay was performed in 1 well per sample, and samples to be considered PRV-positive had Ct values up to 39.9 and with an exponential curve; Ct values between 40 and 45 were considered suspicious, and a sample was negative if there was no Ct value. The analytical sensitivity of the RT-qPCR assay for PRV was previously determined through serial 10-fold dilutions of an in-vitro transcribed RNA (cRNA) of PRV segment L1 cDNA clone L1-C [6] from 10^11^ to 10^0^ copies/μl, and had a detection limit of 1 copy. The standard curve based on the serial 10-fold dilutions in 4 replicates at each dilution had a slope of − 3.437, and the Efficiency was 1.954. The cut-off was adjusted to the minimum copy number detected by the assay at Ct value of 40.0 [39]. The positive control for the PRV RT-qPCR assay was the cRNA used at 10^− 4^ dilution, which gives a low Ct value (~ Ct of 20). The negative control (no-template-control) was water. Ct values of individual tissue samples from the farmed Atlantic salmon, as tested, were visualized through a box plot showing the median and the quartiles of the different tissue samples. One Way Analysis of variance (ANOVA) [40] was used to analyze differences in Ct values among the different tissue samples.

### Conventional RT-PCR and sequence analysis of PRV S1 segment

Selected samples with positive Ct values in the PRV RT-qPCR assay were tested in conventional RT-PCR targeting the full-length genome segment S1 with the PCR primer pair and reaction conditions as previously described [6]. The PCR products were cloned into the pCRII vector using a TOPO TA cloning kit (Invitrogen) in preparation for nucleotide sequencing. Plasmid DNA for sequencing was prepared as described before [41]. DNA sequencing was performed as previously described [42] by ACGT Corporation (Toronto, Ontario, Canada). DNA sequencing was done on plasmid DNA containing the cloned RT-PCR products obtained from reactions using total RNA from tissue samples. Similarity analysis of the obtained DNA sequences was performed using BLAST programs available via the National Center for Biotechnology Information [43]. Analysis to identify putative ORFs and their predicted amino acid sequences was conducted using the Sequence Manipulation suite, version 2 [44].

### Phylogenetic analyses

All PRV S1 segment sequences used in the phylogenetic analyses are described in an additional file in more detail (see Additional file 1: Table S1). The PRV sequences were obtained from selected samples from the escaped farmed Atlantic salmon from the Deepwater Bay, Cypress Island Facility in Puget Sound, Washington State (13 Washington State samples and 2 BC-Canada samples), and from the sample of market-bought fish labeled “Product of Iceland” obtained from a retail store in Redmond, Washington State. A PRV S1 segment sequence WFRC Case-18_01_Sample 7 obtained from separate samples by Western Fisheries Research Centre (WFRC) was included in the analysis. All other PRV sequences were obtained from the GenBank Database [45]. Sequences were processed using ClustalX 2.1 [46]. The multiple sequence alignment was manually verified and adjusted to reach high quality alignment. The phylogenetic trees were generated when positions with gaps were excluded and corrections for multiple substitutions were used. Bootstrapping was performed for 1000 times. In all cases, only the bootstrapping supports higher than 70% were noted. To verify the evolution direction, outgroup sequences were used to determine the root of the phylogenetic trees.

### Statistical analysis: estimation of the total number of escaped salmon that were PRV-positive based on the results of sample analysis

Clark et al. [28] estimated that of the 305,000 farmed salmon in the Deepwater Bay net pen at the time of the catastrophic failure a median number of 253,000 escaped to the marine waters of Bellingham Bay, nearby rivers, and beyond. To estimate the probability that the total number of escaped fish that were infected with PRV was K, given that a total of 253,000 fish escaped, we used our 27 random samples tested for PRV and the 4 fish sampled by Washington Department of Fish and Wildlife as reported in Clark et al. ([28], pp.98), all of which tested positive for PRV (total = 31) in a Bayesian analysis using a beta-binomial likelihood (equation [1]).

(1) P(K|N, S, R) ~ BBN(N, S, R)

where K = the total number infected with PRV, N, the total number escaped (from which samples were obtained (253,000), S = the total number of (random) samples tested for PRV (31), and R = the total number of samples (S) that tested positive for PRV (31), where BBN is the beta-binomial likelihood, N, S, and R are known and K is the unknown to be estimated. The purpose of this analysis was to help estimate the threat that this large escape may pose to wild Pacific salmon and steelhead.

## Ethical approval

Washington State fish samples from fishery captures near the damaged net pen were authorized by the Department of Natural Resources for the express purpose of “cleaning up” farmed Atlantic salmon that escaped from the pen.

British Columbia-Canada fish samples were collected via angling and gillnetting under British Columbia Provincial licenses and from aboriginal food fisheries.

The in-vitro work in this study was approved by the Biosafety Committee of the University of Prince Edward Island.

## Results and discussion

### Population structure of the escaped farmed Atlantic salmon cohort

The weight and fork length of the Atlantic salmon caught by fishermen in Washington State and BC-Canada ranged from 1.8–5.4 kg and 62–77 cm, respectively. This is the approximate size range of the Atlantic salmon reported in the Deepwater Bay net pen facility prior to the escape ([28], pp.86–7).

### Screening for PRV by RT-qPCR in escaped farmed Atlantic salmon

Twenty-eight escaped farmed Atlantic salmon, 23 from Washington State and 5 from British Columbia, and 1 market-bought fish labeled “Product of Iceland” were tested for PRV RNA. For the Washington State fish, tissue samples including gill, heart, liver, spleen or head kidney and eggs (from two fish) were preserved separately and tested individually, whereas samples of gill, heart, spleen, liver, and head kidney were pooled for each of the BC-Canada fish (Table 1). In total, 74 samples were tested for PRV using real-time RT-PCR with TaqMan probe for PRV segment L1 [6] and confirmatory sequencing of PRV segment S1 was done on a subset from these RT-qPCR positive samples. The Ct values for the ELF-1α gene for all samples ranged from 16.91 ± 0.12 to 23.86 ± 0.24, with all samples except ten (from 9 fish) having a Ct value of ≤22.0, which indicated acceptable sample RNA quality [38]. In our experience, the RT-qPCR assay for PRV works well even for samples with ELF-1α Ct values as high as 26.0.

Table 1 summarizes the Ct values of the PRV segment L1 as an indication of PRV load for all samples. All the farmed Atlantic salmon analyzed from Washington State and BC-Canada were positive for PRV (i.e., contained PRV sequences of genome segment L1), as did the purchased sample marketed as “Product of Iceland”. Only one sample of gill tissue from an otherwise PRV-positive fish (WFC-2011-13) was PRV-negative. All 16 samples tested by conventional RT-PCR targeting full-length PRV segment S1 were also positive and generated high quality DNA sequence, confirming PRV infection of the fish that escaped from the Deepwater Bay, Cypress Island facility.

**Fig. 2:**
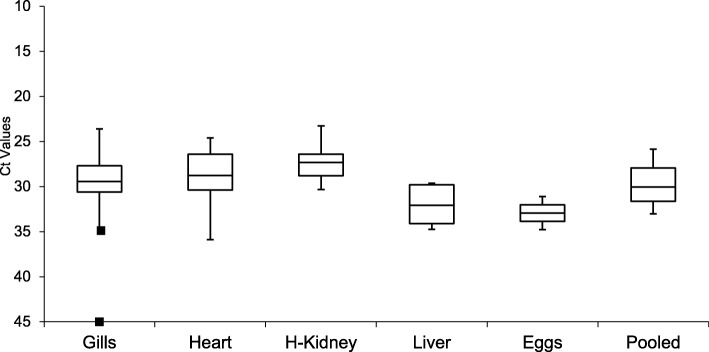
Box plot showing the Ct value distribution of each tissue sample tested in the PRV RT-qPCR assay. Gills: *n* = 21, Heart: n = 21, H-Kidney (Head Kidney): *n* = 17, Liver: *n* = 4, Eggs: n = 2, Pooled (tissue pool of gill, heart, spleen, liver, and head kidney): *n* = 5. The middle lines in each box show the median, and the boxes reflect the quartiles. The error bars indicate the maximum and minimum values within ±1.5 interquartile range of the Ct value distribution. The square (■) represents the minimum and maximum (gills) outliers; the maximum outlier with “No Ct”

The distribution of Ct values among the different tissues tested in the PRV RT-qPCR assay is shown as a box plot (Fig. 2). Outliers were only evident in the gill samples of WFC-2011-14 (35.60 Ct) and WFC-2011-13 (0 Ct). Both fish had generally high Ct values in corresponding heart and head kidney/liver samples (Table 1). One Way Analysis of variance (ANOVA) [40] showed that differences in Ct values among the different tissue samples were not significant (*p* = 0.1121), which ruled out cross-contamination among samples. The Ct values for the egg samples, WFC-2011-111 and WFC-2011-112, were 31.10 and 34.78, which was higher than for the corresponding heart samples (Table 1). Egg sample WFC-2011-111 (31.10 Ct) contained enough PRV genetic material to yield full-length S1 sequence, suggesting that the egg was infected with PRV and not simply contaminated with trace amounts of virus during handling. PRV-positive WFC-2011-112-egg sample (34.78 Ct), did not yield a product in conventional RT-PCR for S1. The Ct values of positive control of *n* = 5 (20.42 ± 0.49) and negative control of n = 5 (0.0) for the PRV RT-qPCR assay were not included in the boxplot of Fig. 2. The Ct value of positive control was significantly different from the fish tissues (*p* = 0.0026).

While eggs from PRV-positive broodfish may test PRV-negative under commercial hatching conditions, 25% of the hatchlings may test positive at very low levels close to the limit of detection and then 100% negative by first feeding [26]. Five out of fourteen hatcheries tested in Norway had PRV-positive pre-smolts, some with Ct values < 25 [3]. Our data confirms previous reports that it is possible to detect PRV RNA in Atlantic salmon eggs. We provide evidence that PRV concentration in eggs may be lower than in other tissues, and that PRV may be vertically transmitted. Since RT-PCR can give false positive results, a second method is normally used to confirm the results. In case of PRV, only RT-PCR methods are available and used for diagnostic testing. In the present study, both RT-qPCR and conventional RT-PCR were used; RT-qPCR targeted L1 segment whereas conventional RT-PCR targeted S1 segment. The positive RT-qPCR results on selected samples were then confirmed by conventional RT-PCR and sequencing of the PCR product, which also allowed for genotyping of the PRV present in samples. The fact that the PRV detected was related to Icelandic PRV shows that the results obtained by our RT-PCR tests are correct.

The 19 Atlantic salmon caught in Washington State that were sampled in between August 27 and December 31 (Table 1) were also tested for infectious haematopoietic necrosis virus (IHNV), viral haemorrhagic septicaemia virus (VHSV), and infectious salmon anemia virus (ISAV), and were RT-PCR negative for these viruses (data not shown). VHSV and ISAV-HPR0 have been reported in Iceland [47, 48].

### Prevalence of PRV in escaped farmed Atlantic salmon

The Atlantic salmon sampled in this study and the four sampled by WDFW reported in Clark et al. [28] (total = 31) can reasonably be considered a random sample from the total estimated number of escapees (253,000) due to the range of fishing gear types applied and non-targeted selection of specimens from the fishermen’s catches. Sampling was opportunistic and did not select for size or condition. These fish can therefore be considered exchangeable with the remaining number of the 253,000 escaped fish that by chance were not sampled. We used these numbers in the beta-binomial likelihood to provide a conservative estimate of the prevalence of PRV in the Deepwater Bay farm equation [2]: (2) K ~ BBN (253,000, 31, 31).

**Fig. 3:**
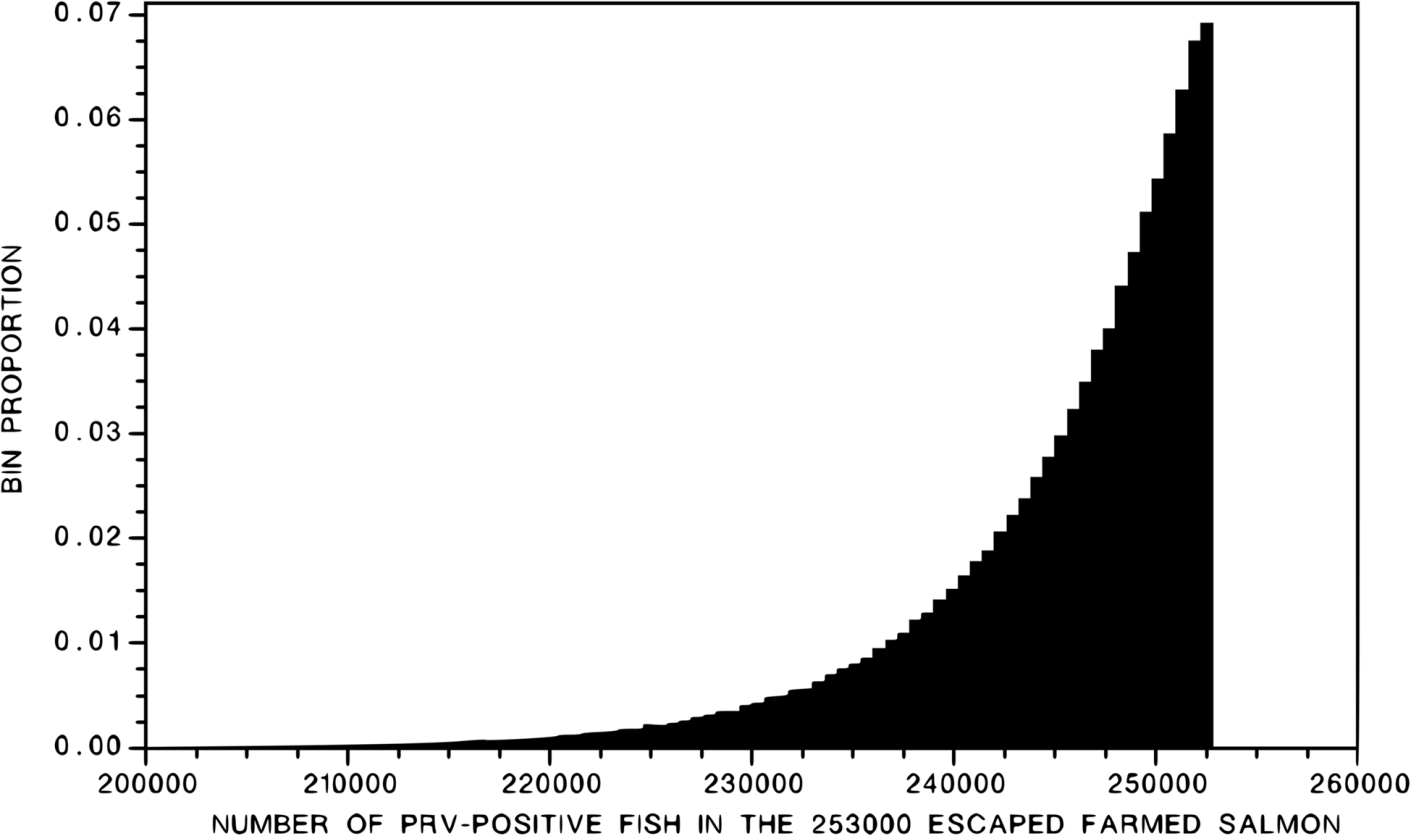
Posterior distribution of the total number of the 253,000 median number of fish estimated to have escaped following the collapse of Deepwater Bay Pen #2 on August 19, 2017, that were PRV-positive based on the results of the 31 random samples all of which tested positive for PRV

The central 95% of the posterior probability distribution of K spanned the interval [230,000, 252,500] (Fig. 3). The mode of distribution is 252,000; the mean is 245,000. Expressing these numbers in terms of the probability (P) that a randomly sampled individual is infected with PRV, the posterior distribution of P spanned the interval [0.91, 0.997], with mean = 0.97 and mode = 0.997. For all practical purposes, all of the fish that escaped were infected with PRV.

### Phylogenetic analysis and sequence diversity of PRV genomic segment S1

**Fig. 4:**
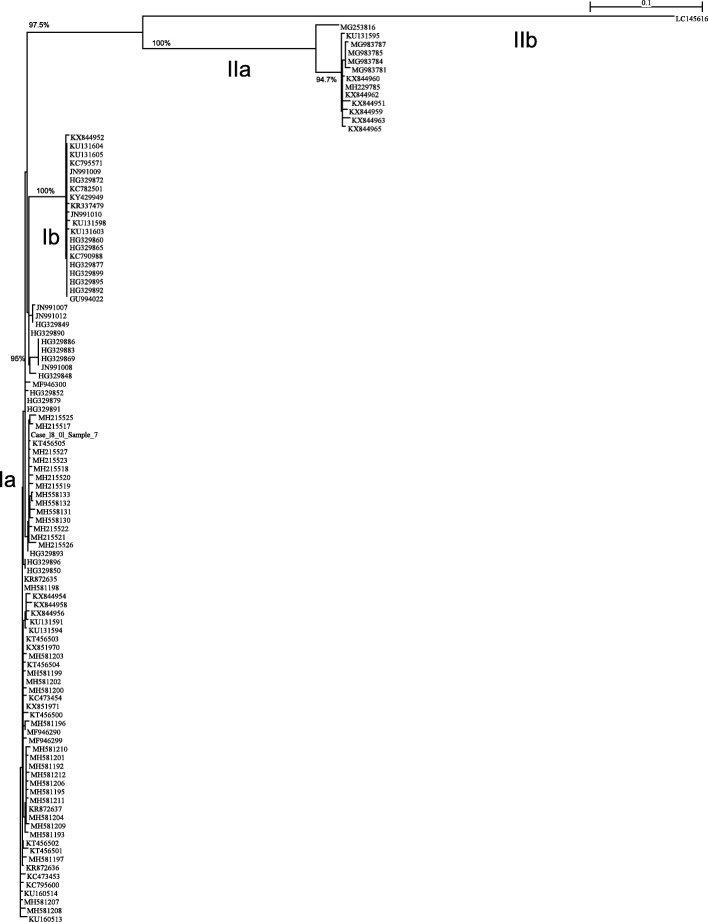
The phylogenetic tree of 107 selected PRV S1 segment sequences was constructed using Maximum Likelihood analysis using PhyML [55]. An outgroup (GenBank accession number: AF059720) was used to determine its root, but the outgroup itself was not included in the tree. The bootstrapping procedure was applied for 1000 times and the branches with 70% or higher bootstrapping support values were marked: each bootstrapping value corresponds to the branch on the same vertical level. The classification of PRV into two genotypes (I and II) and four sub-genotypes (Ia, Ib, IIa, and IIb) is also shown. The PRV S1 segment sequences obtained in this study from the samples of escaped farmed Atlantic salmon and the market-bought fish labeled “Product of Iceland” are highlighted in yellow

Prior to this study, there was only one Icelandic PRV S1 sequence in the GenBank, Accession # KT456505, PRV isolate VT01212014–09 from market-bought fish in BC-Canada labeled “Product of Iceland” [25]. A total of 15 full-length sequences of PRV S1 segment were obtained from the samples of farmed Atlantic salmon tested in this study. Full-length sequence was also obtained from the sample of market-bought fish labeled “Product of Iceland” obtained from a retail store in Redmond, Washington State. We used both Maximum Likelihood and Neighbor Joining methods in phylogenetic analysis and found that they both produced similar trees. The basic sub-genotypes Ia, Ib, and genotype II [5] are all conserved in both trees. It should be noted that the two groups of sub-genotype Ia in the Maximum Likelihood trees are very close and should be considered as one group although visually they appear apart from each other. Also, the bootstrapping values in the Maximum Likelihood trees are very similar to those in the Neighbor Joining trees. Here we show only the trees generated using Maximum Likelihood method. Sequence analyses showed the 16 PRV segment S1 sequences belong to PRV sub-genotype Ia. A phylogenetic tree of these sequences including PRV S1 segment sequence WFRC Case-18_01_Sample 7 obtained from separate samples by WFRC, and selected PRV S1 segment sequences available in the GenBank database (as indicated in Additional file 1: Table S1) is shown in Fig. 4. Thus 107 PRV S1 segment sequences out of the 175 sequences analyzed in this study are included in this phylogenetic tree. All the 175 PRV S1 segment sequences are shown in the phylogenetic tree in an additional file in more detail (see Additional file 2: Figure S1). Although the root of each tree was determined using an outgroup sequence, we did not include the outgroup itself in Fig. 4 and Figure S1, in order to focus on the portion that matters to this analysis. All sequences from the escaped farmed Atlantic salmon fit well inside PRV sub-genotype Ia, and are very similar to GenBank Accession numbers KT456505 (PRV isolate VT01212014–09 from market-bought fish in BC-Canada labeled “Product of Iceland” [25] and HG329893 (Norwegian PRV isolate “195 Aaroy 2007 Wild” [4]). Thus, the PRV strains present in the escaped farmed Atlantic salmon were very similar to PRV in farmed Atlantic salmon in Iceland, the reported country of origin of the Atlantic salmon eggs used in commercial aquaculture in Washington State. This finding of Icelandic PRV in egg samples taken from the escapees in Washington State completes the infection cycle of PRV in farmed Atlantic salmon. It is also the first direct evidence of international movement of PRV via Atlantic salmon eggs.

The sub-genotype grouping in Additional file 2: Figure S1, which includes all PRV isolates, is contrary to the observations by DiCicco et al. [13] that there are some sequences that lie between sub-strains (e.g., JN991012.1, JN991007.1) and therefore the distinction into PRV-1a and 1b is not helpful. It is clearly evident in Additional file 2: Figure S1 and Fig. 4 that Norwegian PRV isolates 1921-S3 (GenBank Accession number JN991007) and 3817-S3 (GenBank Accession number JN991012) of Løvoll et al. [3] belong to sub-genotype Ia, which is distinct from the Norwegian PRV isolate Salmo/GP-2010/NOR (GenBank Accession number GU994022) [1] utilized by DiCicco et al. [13] as the reference sequence PRV-1a but belongs to sub-genotype Ib. At the time of writing this paper only two of these PRV-1a sequences, isolates B5690 and B7274 (GenBank Accession numbers KX851970 and KX851971, respectively) [10] were available in the GenBank Database, and both belong to sub-genotype Ia (Fig. 4). The Norwegian PRV isolate NOR2012-V3621 (GenBank Accession number KY429949) used to reproduce HSMI in an Atlantic salmon challenge study [49] belongs to sub-genotype Ib (Fig. 4). Additional detailed data are in Additional file 1: Table S1 and Additional file 2: Figure S1. Thus, this phylogenetic analysis shows that the second genotype, genotype II, identified by Godoy et al. [7] among PRV isolates from Norway and Chile also includes PRV-2 found in Japan [8] and several PRV isolates (designated PRV-3) found in rainbow trout in Norway and several European countries and in brown trout in Italy [16] and France (Bigarre 2016, unpublished observations) and in rainbow trout [17] and Coho salmon in Chile [18]. We therefore further provide strong support for classification of PRV into two genotypes (I and II) and four sub-genotypes (Ia, Ib, IIa, and IIb).

## Conclusions

Close to 100% of the Atlantic salmon collected following the escape of 253,000 Atlantic salmon from a farm in Puget Sound, Washington State, were infected with a strain of PRV that appears to have originated from Iceland. The PRV sequences from these farmed Atlantic salmon were very similar to PRV isolates from the hatchery in Iceland purported to be the origin of the Atlantic salmon used in commercial aquaculture in Washington State. This study emphasizes the need to screen Atlantic salmon broodstock for PRV, particularly where used to supply widely distributed Atlantic salmon eggs and where Atlantic salmon are farmed among wild Pacific salmon populations.

## Additional files

Additional file 1: **Table S1.** Piscine reovirus segment S1 nucleotide sequences analysed in this study [1, 3, 4, 6–8, 10, 16–18, 24, 49–54]. (DOC 298 kb)

Additional file 2: **Figure S1.** The phylogenetic tree of all PRV S1 segment sequences analyzed in this study (175 sequences) was constructed using Maximum Likelihood analysis using PhyML [55]. An outgroup (GenBank accession number: AF059720) was used to determine its root, but the outgroup itself was not included in the tree. The bootstrapping procedure was applied for 1000 times and the branches with 70% or higher bootstrapping support values were marked: each bootstrapping value corresponds to the branch on the same vertical level. The classification of PRV into two genotypes (I and II) and four sub-genotypes (Ia, Ib, IIa, and IIb) is also shown. The PRV S1 segment sequences obtained in this study from the samples of escaped farmed Atlantic salmon and the market-bought fish labeled “Product of Iceland” are highlighted in yellow (PDF 31 kb)

## Abbreviations

#: Number
ANOVA: One Way Analysis of variance
AVC: Atlantic Veterinary College
BBN: Beta-binomial likelihood
BC: British Columbia
BC-Canada: British Columbia-Canada
BLAST: Basic local alignment search tool
cDNA: Complimentary DNA
CFIA: Canadian Food Inspection Agency
cm: Centimeters
Cp: Crossing point
cRNA: In-vitro transcribed RNA
Ct: Threshold cycle
DFW: Washington State Department of Wildlife
DNA: Deoxy ribonucleic acid
DNR: Washington State Department of Natural Resources
DOE: Washington State Department of Ecology
EIBS: Erythrocytic inclusion body syndrome
ELF-1α: Elongation factor 1 alpha
Equation [1]: (1) P(K|N, S, R) ~ BBN(N, S, R)
Equation [2]: (2) K ~ BBN
HSMI: Heart and skeletal muscle inflammation
HSMI-like: Heart and skeletal muscle inflammation-like
ID: Identintification number
IHNV: Infectious haematopoietic necrosis virus
Inc: Incorporated
ISAV: Infectious salmon anemia virus
ISAV-HPR0: Infectious salmon anemia virus with no deletion in highly pleomorphic region
ISRT: Idiopathic syndrome of rainbow trout
K: Total number of fish infected with PRV
kg: Kilograms
N: Total number of fish escaped (from which samples were obtained)
^o^C: Centigrade
ORFs: Open reading frames
p or P: Probability
P: Probability
pCRII: pCR™-TOPO® plasmid with T7 and SP6 promoters
pp.: Page numbers
PRV I, PRV II: Different genotypes of Piscine orthoreovirus
PRV: Piscine orthoreovirus
PRV-1, PRV-2, PRV3, PRV-3b: Different isolates of Piscine orthoreovirus
PRV-Ia (or PRV Ia), PRV-Ib, PRV-Ic, Ia, Ib, IIa, IIb: Different subgenotypes of Piscine orthoreovirus
R: Total number of fish (S) that tested positive for PRV
RNA: Ribonucleic acid
RT-PCR: Reverse transcriptase polymerase chain reaction
RT-qPCR: Real time quantitative Reverse transcriptase polymerase chain reaction
S: Total number of (random) fish tested for PRV
Segment L1 or Seg. L1: Large segment 1
Segment S1 or Seg. S1: Small segment 1
TOPO TA: DNA cloning technique of PCR product into pCR™-TOPO® plasmid in the presence of topoisomerase
USA: United States of America
VHSV: Viral haemorrhagic septicaemia virus
WA: Washington State
WFC: Wild Fish Conservancy
WFRC: Western Fisheries Research Centre
