# Correction to: Piscine orthoreovirus sequences in escaped farmed Atlantic salmon in Washington and British Columbia

**DOI:** 10.1186/s12985-019-1166-0

**Published:** 2019-05-07

**Authors:** Molly J. T. Kibenge, Yingwei Wang, Nick Gayeski, Alexandra Morton, Kurt Beardslee, Bill McMillan, Frederick S. B. Kibenge

**Affiliations:** 1 Department of Pathology and Microbiology, Atlantic Veterinary College, University of Prince Edward Island, 550 University Ave, Charlottetown, P.E.I C1A 4P3 Canada; 2 School of Mathematical and Computational Sciences, University of Prince Edward Island, 550 University Ave, Charlottetown, P.E.I C1A 4P3 Canada; 3 Wild FishConservancy, PO Box 402, 15629 Main St. NE, Duvall, WA 98019 USA; 4 RaincoastResearch Society, Box 399, Sointula, BC V0N 3E0 Canada

**Correction to: Virology Journal (2019) 16:41**

**https://doi.org/10.1186/s12985-019-1148-2**

In the original publication of the article [1], as the quotation below was included without specific permission from Dr. Gary Marty, which is against the *Virology Journal* guidelines for the citation of unpublished data, all authors request to delete it from their article.

“*The largest number of farmed Atlantic salmon tested for PRV in BC-Canada found PRV in approximately 80% of 146 pooled samples from 539 Atlantic salmon tested in 2010 (Marty and Bidulka, 2013, unpublished observations).*”
