# Supplementary material for: Piscine orthoreovirus sequences in escaped farmed Atlantic salmon in Washington and British Columbia
Source: Virol J. 2019 Apr 2;16:41. doi: 10.1186/s12985-019-1148-2 (PMC6444584; doi:10.1186/s12985-019-1148-2)
Supplement: Supplementary file 1 — Table S1. Piscine reovirus segment S1 nucleotide sequences analysed in this study [1, 3, 4, 6–8, 10, 16–18, 24, 49–54]. (DOC 298 kb) [file 12985_2019_1148_MOESM1_ESM.doc]

**Table S1:** Piscine orthoreovirus segment S1 nucleotide sequences analyzed in this study

| **PRV isolate ID** | **Country** | **GenBank Accession number** | **Sub-genotype (this study)^1^** | **Figure 4 (this study)** | **Reference** | **Year** |
| --- | --- | --- | --- | --- | --- | --- |
| VT02292012-163 | Canada | KC473452 | Ia |  | [6] | 2012 |
| VT06062012-358 | Canada | KC473453 | Ia | YES | [6] | 2012 |
| VT06202012-371 | Canada | KC473454 | Ia | YES | [6] | 2012 |
| VT02292012-167 | Canada | KC795599 | Ia |  | [6] | 2012 |
| VT03202012-196 | Canada | KC795600 | Ia | YES | [6] | 2012 |
| VT03202012-209 | Canada | KC795601 | Ia |  | [6] | 2012 |
| VT09122012-755 | Canada | KT456500 | Ia | YES | [51] | 2011 |
| VT09192013-402 | Canada | KT456501 | Ia | YES | [51] | 2013 |
| VT09192013-408 | Canada | KT456502 | Ia | YES | [51] | 2013 |
| VT01212014-03 | Canada | KT456503 | Ia | YES | [51] | 2014 |
| VT01212014-04 | Canada | KT456504 | Ia | YES | [51] | 2014 |
| VT01212014-09 | Iceland | KT456505 | Ia | YES | [51] | 2014 |
| VT01292015-09 | Canada | KU160513 | Ia | YES | [51] | 2015 |
| VT07222015-106 | Canada | KU160514 | Ia | YES | [51] | 2015 |
| VT07222015-107 | Canada | KU160515 | Ia |  | [51] | 2015 |
| VT10092015-122 | Canada | MF946290 | Ia | YES | [52] | 2015 |
| VT02162017-59 | Norway | MF946299 | Ia | YES | [52] | 2017 |
| VT03022017-69 | Canada | MF946300 | Ia | YES | [52] | 2017 |
| VT05252017-236 | Canada | MH581192 | Ia | YES | [53] | 2017 |
| VT06092017-347 | Canada | MH581193 | Ia | YES | [53] | 2017 |
| VT06092017-348 | Canada | MH581194 | Ia |  | [53] | 2017 |
| VT10042017-382 | Canada | MH581195 | Ia | YES | [53] | 2017 |
| VT10042017-383 | Canada | MH581196 | Ia | YES | [53] | 2017 |
| VT10252017-421 | Canada | MH581197 | Ia | YES | [53] | 2017 |
| VT10252017-422 | Canada | MH581198 | Ia | YES | [53] | 2017 |
| VT11302017-453 | Canada | MH581199 | Ia | YES | [53] | 2017 |
| VT11302017-454 | Canada | MH581200 | Ia | YES | [53] | 2017 |
| VT03022018-66 | Canada | MH581201 | Ia | YES | [53] | 2018 |
| VT03022018-67 | Canada | MH581202 | Ia | YES | [53] | 2018 |
| VT03022018-68 | Canada | MH581203 | Ia | YES | [53] | 2018 |
| VT03262018-75-27 | Canada | MH581204 | Ia | YES | [53] | 2018 |
| VT03262018-77 | Canada | MH581205 | Ia |  | [53] | 2018 |
| VT03262018-83 | Canada | MH581206 | Ia | YES | [53] | 2018 |
| VT03262018-84 | Canada | MH581207 | Ia | YES | [53] | 2018 |
| VT03262018-88 | Canada | MH581208 | Ia | YES | [53] | 2018 |
| VT04062018-105 | Canada | MH581209 | Ia | YES | [53] | 2018 |
| VT04062018-106 | Canada | MH581210 | Ia | YES | [53] | 2018 |
| VT04062018-108 | Canada | MH581211 | Ia | YES | [53] | 2018 |
| VT04062018-109 | Canada | MH581212 | Ia | YES | [53] | 2018 |
| VT01192018-07 | Iceland | MH215517 | Ia | YES | This study | 2017 |
| VT01192018-16 | Iceland | MH215518 | Ia | YES | This study | 2017 |
| VT01192018-21 | Iceland | MH215519 | Ia | YES | This study | 2017 |
| VT01192018-26 | Iceland | MH215520 | Ia | YES | This study | 2017 |
| VT01192018-33 | Iceland | MH215521 | Ia | YES | This study | 2017 |
| VT01192018-43 | Iceland | MH215522 | Ia | YES | This study | 2017 |
| VT01192018-48 | Iceland | MH215523 | Ia | YES | This study | 2017 |
| VT01192018-52 | Iceland | MH215524 | Ia |  | This study | 2017 |
| VT01192018-53 | Iceland | MH215525 | Ia | YES | This study | 2017 |
| VT10252017-405 | Iceland | MH215526 | Ia | YES | This study | 2017 |
| VT11022017-423 | Iceland | MH215527 | Ia | YES | This study | 2017 |
| VT04202018-119 | Iceland | MH558129 | Ia |  | This study | 2018 |
| VT04202018-121 | Iceland | MH558130 | Ia | YES | This study | 2018 |
| VT04202018-123 | Iceland | MH558131 | Ia | YES | This study | 2018 |
| VT04202018-124 | Iceland | MH558132 | Ia | YES | This study | 2018 |
| VT04202018-129 | Iceland | MH558133 | Ia | YES | This study | 2018 |
| Case_18_01_Sample 7 | Iceland | not in GenBank | Ia | YES | WFRC^2^ |  |
| BCinoc3 | Canada | KR872635 | Ia | YES | [54] | 2012 |
| BCinoc12_13 | Canada | KR872636 | Ia | YES | [54] | 2013 |
| BC361_14 | Canada | KR872637 | Ia | YES | [54] | 2014 |
| B7274 | Canada | KX851971 | Ia | YES | [10] | 2013 |
| B5690 | Canada | KX851970 | Ia | YES | [10] | 2013 |
| 5433-S3 | Norway | JN991006 | Ia |  | [3] | 2012 |
| 1921-S3 | Norway | JN991007 | Ia | YES | [3] | 2012 |
| 9326-S3 | Norway | JN991008 | Ia | YES | [3] | 2012 |
| 3817-S3 | Norway | JN991012 | Ia | YES | [3] | 2012 |
| 35 Bjoreio | Norway | HG329842 | Ia |  | [4] | 2009 |
| 45 Eira | Norway | HG329843 | Ia |  | [4] | 2009 |
| 131 Gaula | Norway | HG329848 | Ia | YES | [4] | 2009 |
| 182 Hestdal | Norway | HG329849 | Ia | YES | [4] | 2009 |
| 187 Hestdal | Norway | HG329850 | Ia | YES | [4] | 2009 |
| 190 Hestdal | Norway | HG329851 | Ia |  | [4] | 2009 |
| 211 Mandal | Norway | HG329852 | Ia | YES | [4] | 2009 |
| 246 Mandal | Norway | HG329854 | Ia |  | [4] | 2009 |
| 284 Stjordal | Norway | HG329858 | Ia |  | [4] | 2009 |
| 307 Stjordal | Norway | HG329859 | Ia |  | [4] | 2009 |
| 407 Vosso | Norway | HG329863 | Ia |  | [4] | 2009 |
| 445 Alta | Norway | HG329868 | Ia |  | [4] | 2008 |
| 470 Drevja | Norway | HG329869 | Ia | YES | [4] | 2008 |
| 517 Eira | Norway | HG329871 | Ia |  | [4] | 2008 |
| 629 Jolstra | Norway | HG329875 | Ia |  | [4] | 2008 |
| 708 Nausta | Norway | HG329876 | Ia |  | [4] | 2008 |
| 842 Vikja | Norway | HG329878 | Ia |  | [4] | 2008 |
| 851 Vikja | Norway | HG329879 | Ia | YES | [4] | 2008 |
| 866 Vikja | Norway | HG329880 | Ia |  | [4] | 2008 |
| 907 Vosso | Norway | HG329881 | Ia |  | [4] | 2008 |
| 909 Vosso | Norway | HG329882 | Ia |  | [4] | 2008 |
| 931 Alta | Norway | HG329883 | Ia | YES | [4] | 2007 |
| 985 Ekso | Norway | HG329885 | Ia |  | [4] | 2007 |
| 987 Ekso | Norway | HG329886 | Ia | YES | [4] | 2007 |
| 989 Ekso | Norway | HG329887 | Ia |  | [4] | 2007 |
| 993 Ekso | Norway | HG329888 | Ia |  | [4] | 2007 |
| 1039 Laerdal | Norway | HG329889 | Ia |  | [4] | 2007 |
| 1062 Mandal | Norway | HG329890 | Ia | YES | [4] | 2007 |
| 1137 Stjordal | Norway | HG329891 | Ia | YES | [4] | 2007 |
| 1195 Aaroy | Norway | HG329893 | Ia | YES | [4] | 2007 |
| 1343 Moelv | Norway | HG329896 | Ia | YES | [4] | 2008 |
| VT12202013_CGA_2013_4 | Chile | KU131591 | Ia | YES | [7] | 2013 |
| VT12202013_CGA_2013_1 | Chile | KU131592 | Ia |  | [7] | 2013 |
| VT12202013_CGA_2013_2 | Chile | KU131593 | Ia |  | [7] | 2013 |
| 2015_CGA_2015_B | Chile | KU131594 | Ia | YES | [7] | 2015 |
| V/P1.1 | Chile | KX844958 | Ia | YES | [17] | 2014 |
| V/P1.2 | Chile | KX844957 | Ia |  | [17] | 2014 |
| V/P2.1 | Chile | KX844956 | Ia | YES | [17] | 2014 |
| V/P2.2 | Chile | KX844955 | Ia |  | [17] | 2014 |
| V/P3.1 | Chile | KX844954 | Ia | YES | [17] | 2014 |
| V/P3.2 | Chile | KX844953 | Ia |  | [17] | 2014 |
| 61 Eira | Norway | HG329844 | Ib |  | [4] | 2009 |
| 81 Etne | Norway | HG329845 | Ib |  | [4] | 2009 |
| 90 Etne | Norway | HG329846 | Ib |  | [4] | 2009 |
| 93 Etne | Norway | HG329847 | Ib |  | [4] | 2009 |
| 220 Mandal | Norway | HG329853 | Ib |  | [4] | 2009 |
| 261 Nidelv | Norway | HG329855 | Ib |  | [4] | 2009 |
| 273 Skibotn | Norway | HG329856 | Ib |  | [4] | 2009 |
| 283 Skjomen | Norway | HG329857 | Ib |  | [4] | 2009 |
| 318 Storelva Holt | Norway | HG329860 | Ib | YES | [4] | 2009 |
| 338 Surna | Norway | HG329861 | Ib |  | [4] | 2009 |
| 350 Surna | Norway | HG329862 | Ib |  | [4] | 2009 |
| 411 Vosso | Norway | HG329864 | Ib |  | [4] | 2009 |
| 412 Vosso | Norway | HG329865 | Ib |  | [4] | 2009 |
| 414 Vosso | Norway | HG329866 | Ib |  | [4] | 2009 |
| 438 Alta | Norway | HG329867 | Ib |  | [4] | 2008 |
| 491 Eira | Norway | HG329870 | Ib |  | [4] | 2008 |
| 522 Ekso | Norway | HG329872 | Ib | YES | [4] | 2008 |
| 555 Fusta | Norway | HG329873 | Ib |  | [4] | 2008 |
| 565 Gaula | Norway | HG329874 | Ib |  | [4] | 2008 |
| 818 Surna | Norway | HG329877 | Ib | YES | [4] | 2008 |
| 982 Eira | Norway | HG329884 | Ib |  | [4] | 2007 |
| 1160 Surna | Norway | HG329892 | Ib | YES | [4] | 2007 |
| 1261 Halsan | Norway | HG329894 | Ib |  | [4] | 2009 |
| 1309 Eidsdal | Norway | HG329895 | Ib | YES | [4] | 2008 |
| 1361 Moelv | Norway | HG329897 | Ib |  | [4] | 2008 |
| 1459 Etne | Norway | HG329898 | Ib |  | [4] | 2010 |
| 1462 Etne | Norway | HG329899 | Ib | YES | [4] | 2010 |
| 1463 Etne | Norway | HG329900 | Ib |  | [4] | 2010 |
| 1469 Etne | Norway | HG329901 | Ib |  | [4] | 2010 |
| 7243-S3 | Norway | JN991009 | Ib | YES | [3] | 2012 |
| 7030-S3 | Norway | JN991010 | Ib | YES | [3] | 2012 |
| 8286-S3 | Norway | JN991011 | Ib |  | [3] | 2012 |
| Salmo/GP-2010/NOR | Norway | GU994022 | Ib | YES | [1] | 2010 |
| 050607 | Norway | KR337479 | Ib | YES | [55] | 2007 |
| NOR2012-V3621 | Norway | KY429949 | Ib | YES | [50] | 2012 |
| CGA337 | Chile | KC782501 | Ib | YES | [6] | 2012 |
| CGA8857 | Chile | KC790988 | Ib | YES | [6] | 2012 |
| CGA280-05 | Chile | KC795571 | Ib | YES | [6] | 2012 |
| 2013_CGA_2013_A | Chile | KU131597 | Ib |  | [7] | 2013 |
| 2013_CGA_2013_C | Chile | KU131598 | Ib | YES | [7] | 2013 |
| VT02182014_CGA_2013_8 | Chile | KU131599 | Ib |  | [7] | 2013 |
| VT02182014_CGA_2013_9 | Chile | KU131600 | Ib |  | [7] | 2013 |
| VT02182014_CGA_2013_10 | Chile | KU131601 | Ib |  | [7] | 2013 |
| VT02182014_CGA_2013_6 | Chile | KU131602 | Ib |  | [7] | 2013 |
| VT02182014_CGA_2013_7 | Chile | KU131603 | Ib | YES | [7] | 2013 |
| 2015_CGA_2015_A | Chile | KU131604 | Ib | YES | [7] | 2015 |
| 2015_CGA_2015_C | Chile | KU131605 | Ib | YES | [7] | 2015 |
| IM/P5.2 | Chile | KX844952 | Ib | YES | [17] | 2015 |
| VT12202013_CGA_2013_3 | Chile | KU131595 | IIa | YES | [7] | 2013 |
| VT12202013_CGA_2013_5 | Chile | KU131596 | IIa |  | [7] | 2013 |
| C10/P1.1 | Chile | KX844965 | IIa | YES | [17] | 2014 |
| C10/P1.2 | Chile | KX844964 | IIa |  | [17] | 2014 |
| C10/P2.1 | Chile | KX844963 | IIa | YES | [17] | 2014 |
| C10/P2.2 | Chile | KX844962 | IIa | YES | [17] | 2014 |
| C10/P3.1 | Chile | KX844961 | IIa |  | [17] | 2014 |
| C10/P3.2 | Chile | KX844960 | IIa | YES | [17] | 2014 |
| C10/P4.2 | Chile | KX844959 | IIa | YES | [17] | 2014 |
| C10/P4.1 | Chile | KX844951 | IIa | YES | [17] | 2014 |
| ADLPRV3 | Chile | MH229785 | IIa | YES | [18] | 2017 |
| IT/17-267 | Italy | MG983784 | IIa | YES | [16] | 2017 |
| IT/17-211.3 | Italy | MG983783 | IIa |  | [16] | 2017 |
| 773 | Germany | MG983787 | IIa | YES | [16] | 2017 |
| G1491 | Scotland | MG983781 | IIa | YES | [16] | 2017 |
| DK/17-18918-13 | Denmark | MG983782 | IIa |  | [16] | 2017 |
| DK/17-18918-6 | Denmark | MG983786 | IIa |  | [16] | 2017 |
| DK/17-18918-1 | Denmark | MG983785 | IIa | YES | [16] | 2017 |
| NOR/060214 | Norway | MG983780 | IIa |  | [16] | 2013 |
| NOR/060214 | Norway | MG253816 | IIa | YES | [16] | 2014 |
| PRV-2 | Japan | LC145616 | IIb | YES | [8] | 2012 |

^1^Genotype classification previously reported in Kibenge *et al.* [6,24] and Godoy *et al.* [7]. Additional details provided in Additional file 2: Figure S1.

^2^WFRC denotes Western Fisheries Research Centre, USGS, Seattle, WA, USA. Collection date of PRV isolate Case_18_01_Sample 7 not provided.
