# Supplementary material for: Piscine orthoreovirus sequences in escaped farmed Atlantic salmon in Washington and British Columbia
Source: Virol J. 2019 Apr 2;16:41. doi: 10.1186/s12985-019-1148-2 (PMC6444584; doi:10.1186/s12985-019-1148-2)
Supplement: Supplementary file 2 — Figure S1. The phylogenetic tree of all PRV S1 segment sequences analyzed in this study (175 sequences) was constructed using Maximum Likelihood analysis using PhyML [55]. An outgroup (GenBank accession number: AF059720) was used to determine its root, but the outgroup itself was not included in the tree. The bootstrapping procedure was applied for 1000 times and the branches with 70% or higher bootstrapping support values were marked: each bootstrapping value corresponds to the branch on the same vertical level. The classification of PRV into two genotypes (I and II) and four sub-genotypes (Ia, Ib, IIa, and IIb) is also shown. The PRV S1 segment sequences obtained in this study from the samples of escaped farmed Atlantic salmon and the market-bought fish labeled “Product of Iceland” are highlighted in yellow (PDF 31 kb) [file 12985_2019_1148_MOESM2_ESM.pdf]

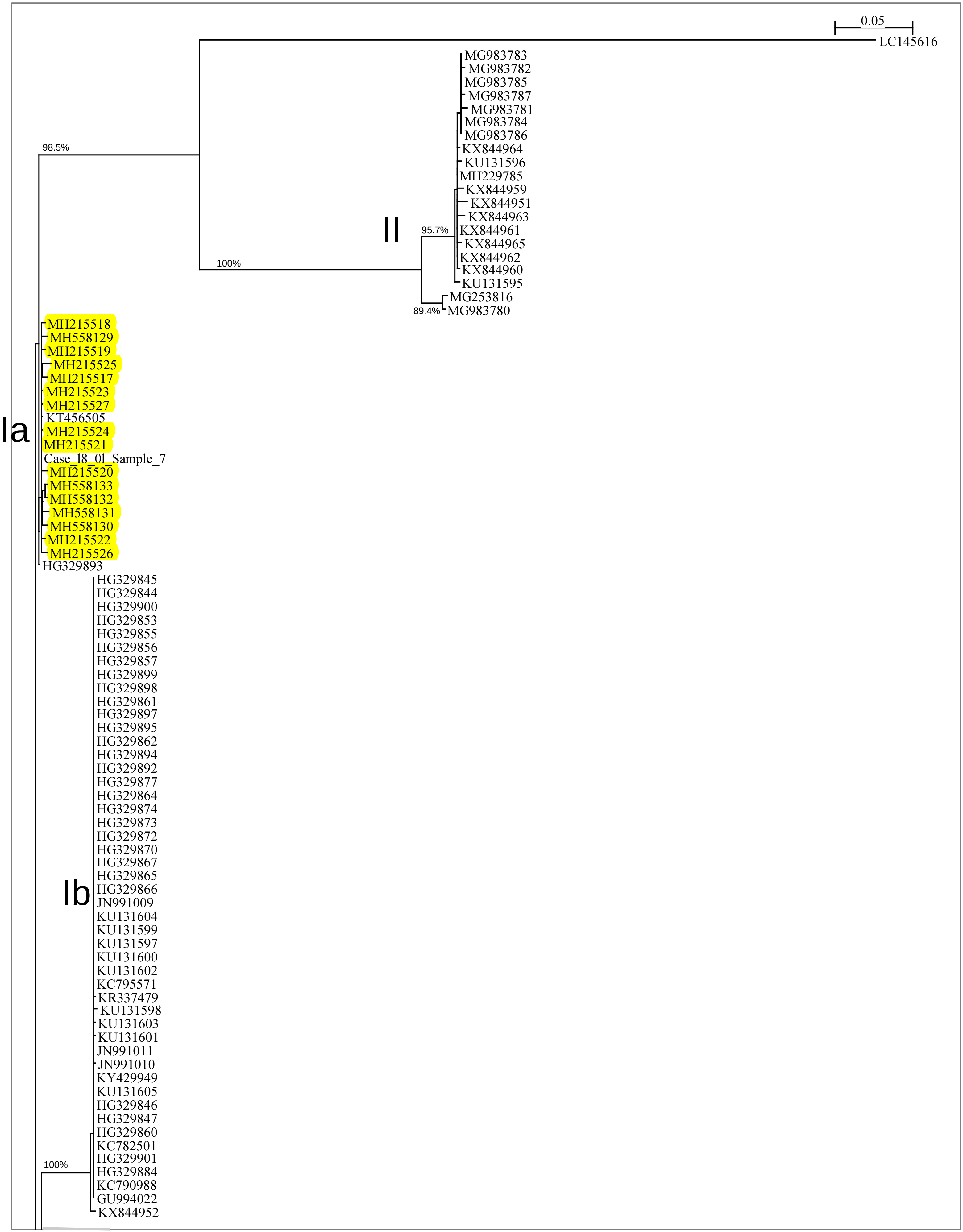
0.05
LC145616
MG983783
MG983782
MG983785
MG983787
MG983781
MG983784
MG983786
KX844964
KU131596
MH229785
KX844959
KX844951
KX844963
KX844961
KX844965
KX844962
KX844960
KU131595
MG253816
MG983780
98.5%
100%
95.7%
89.4%
II
MH215518
MH558129
MH215519
MH215525
MH215517
MH215523
MH215527
KT456505
MH215524
MH215521
Case_18_01_Sample_7
MH215520
MH558133
MH558132
MH558131
MH558130
MH215522
MH215526
HG329893
Ia
HG329845
HG329844
HG329900
HG329853
HG329855
HG329856
HG329857
HG329899
HG329898
HG329861
HG329897
HG329895
HG329862
HG329894
HG329892
HG329877
HG329864
HG329874
HG329873
HG329872
HG329870
HG329867
HG329865
HG329866
JN991009
KU131604
KU131599
KU131597
KU131600
KU131602
KC795571
KR337479
KU131598
KU131603
KU131601
JN991011
JN991010
KY429949
KU131605
HG329846
HG329847
HG329860
KC782501
HG329901
HG329884
KC790988
GU994022
KX844952
Ib
100%

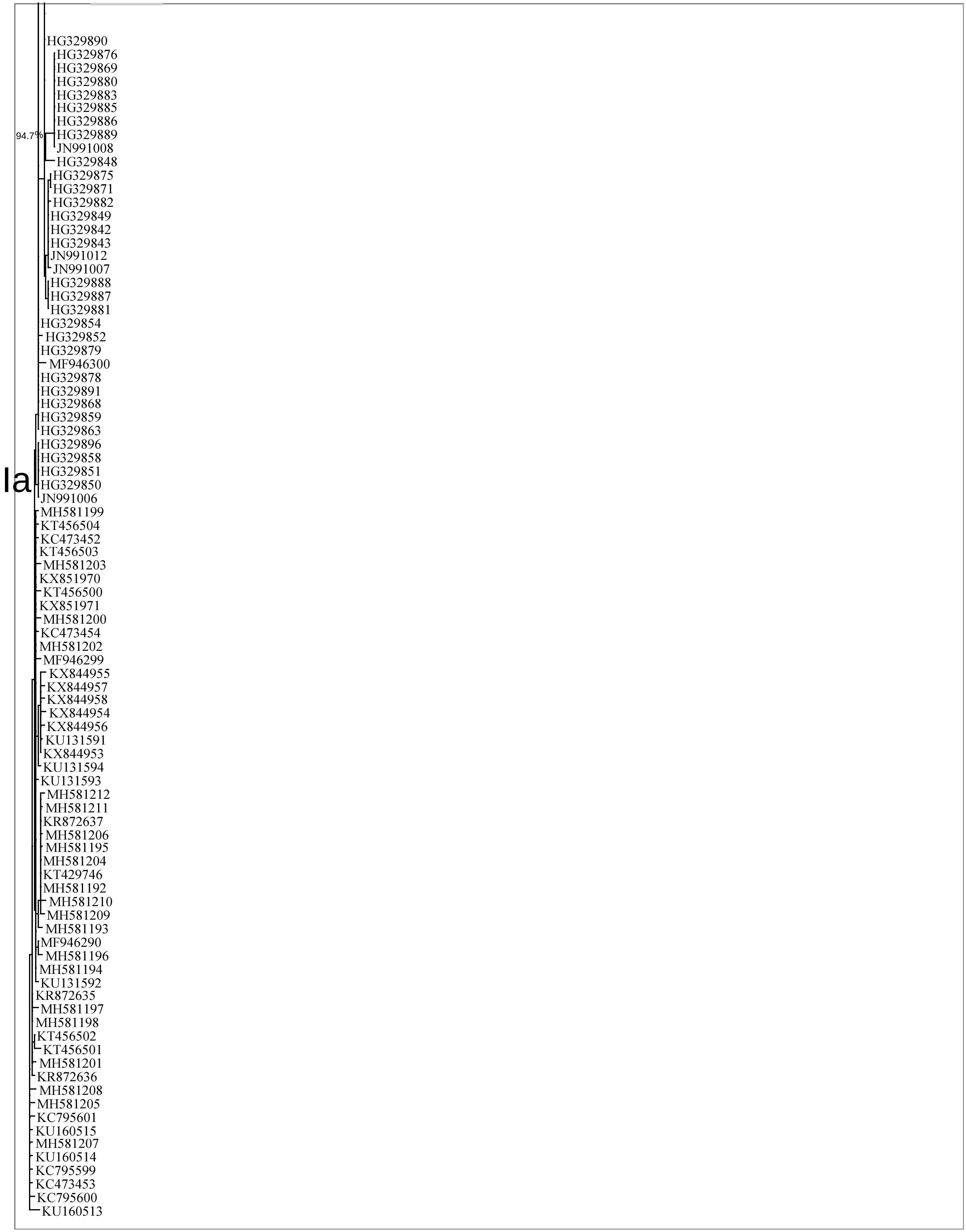
94.7%
Ia
HG329890
HG329876
HG329869
HG329880
HG329883
HG329885
HG329886
HG329889
JN991008
HG329848
HG329875
HG329871
HG329882
HG329849
HG329842
HG329843
JN991012
JN991007
HG329888
HG329887
HG329881
HG329854
HG329852
HG329879
MF946300
HG329878
HG329891
HG329868
HG329859
HG329863
HG329896
HG329858
HG329851
HG329850
JN991006
MH581199
KT456504
KC473452
KT456503
MH581203
KX851970
KT456500
KX851971
MH581200
KC473454
MH581202
MF946299
KX844955
KX844957
KX844958
KX844954
KX844956
KU131591
KX844953
KU131594
KU131593
MH581212
MH581211
KR872637
MH581206
MH581195
MH581204
KT429746
MH581192
MH581210
MH581209
MH581193
MF946290
MH581196
MH581194
KU131592
KR872635
MH581197
MH581198
KT456502
KT456501
MH581201
KR872636
MH581208
MH581205
KC795601
KU160515
MH581207
KU160514
KC795599
KC473453
KC795600
KU160513
